# Coping with Oxidative Stress in Reproductive Pathophysiology and Assisted Reproduction: Melatonin as an Emerging Therapeutical Tool

**DOI:** 10.3390/antiox12010086

**Published:** 2022-12-30

**Authors:** Patricia Cosme, Ana B. Rodríguez, María Garrido, Javier Espino

**Affiliations:** Neuroimmunophysiology and Chrononutrition Research Group, Department of Physiology, Faculty of Science, University of Extremadura, 06006 Badajoz, Spain

**Keywords:** oxidative stress, melatonin, infertility, assisted reproductive techniques, oocyte quality, sperm quality, cryopreservation

## Abstract

Infertility is an increasing global public health concern with socio-psychological implications for affected couples. Remarkable advances in reproductive medicine have led to successful treatments such as assisted reproductive techniques (ART). However, the search for new therapeutic tools to improve ART success rates has become a research hotspot. In the last few years, pineal indolamine melatonin has been investigated for its powerful antioxidant properties and its role in reproductive physiology. It is considered a promising therapeutical agent to counteract the detrimental effects associated with oxidative stress in fertility treatments. The aim of the present narrative review was to summarize the current state of the art on the importance of melatonin in reproductive physiology and to provide a critical evaluation of the data available encompassing basic, translational and clinical studies on its potential use in ART to improve fertility success rates.

## 1. Introduction

Infertility is an increasing global public health issue that affects up to 16% of couples of reproductive age [1]. According to the World Health Organization (WHO), infertility is a disease of the reproductive system defined by the failure to achieve a clinical pregnancy after 12 months of regular, unprotected sexual intercourse [2]. Half of infertility cases are due to female factors [3], which are generally attributed to hormonal, functional or anatomical dysfunction of the organs of the reproductive tract [4]. Moreover, the age of the female is of great importance because of its physiological and genetic influence on conception, which includes a reduced ovarian follicular pool, perturbations in ovulation and increased meiotic errors within the oocyte [5]. As for male factors, these are responsible for 20–30% of infertility cases. The most important factors are hormonal deficits, physical causes, sexually transmitted problems, and genetic factors. Nevertheless, the origin of about 40% of male infertility is unknown [3].

**Figure 1:**
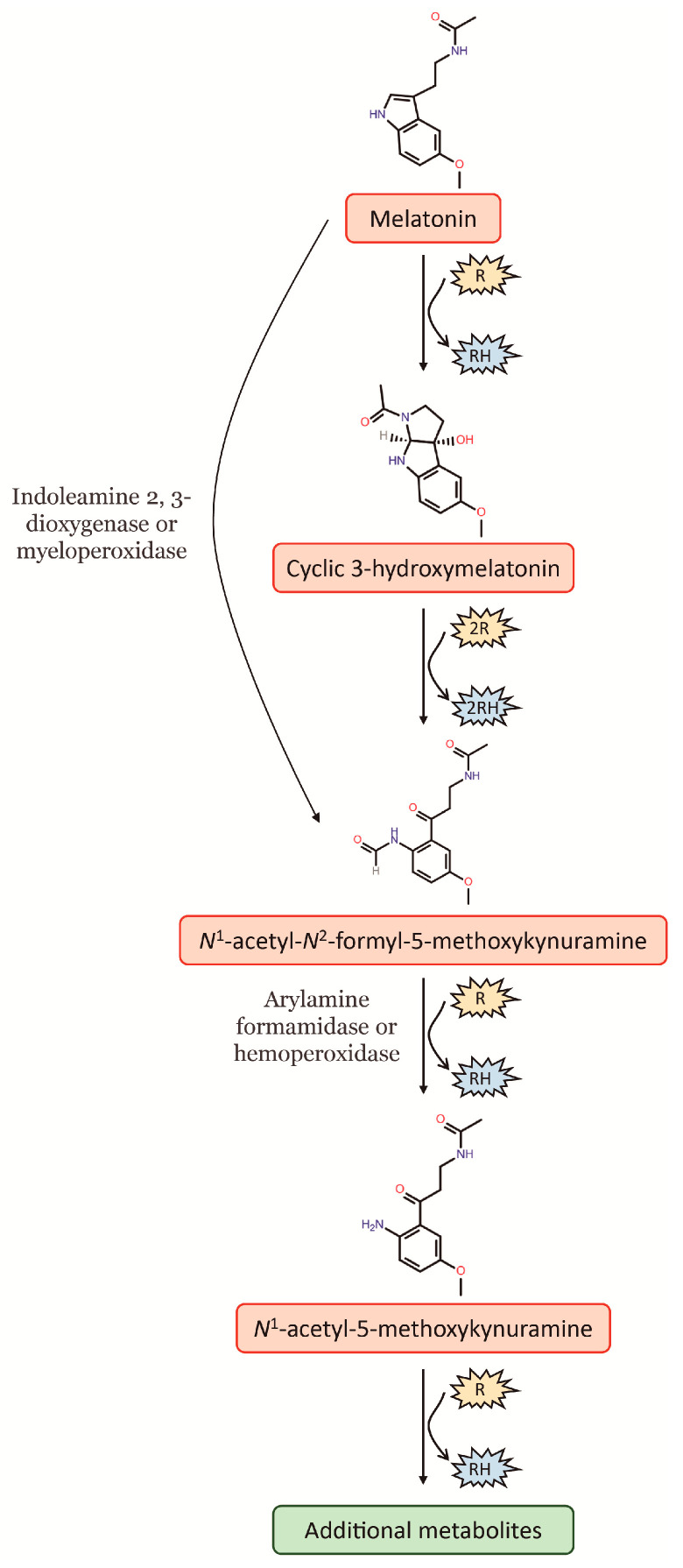
Cascade reaction of melatonin interaction with free radicals and its main metabolites. R: free radical; RH: reduced agent.

Melatonin is well-known for its potent antioxidant properties, as it can remove free radicals and convert reactive oxygen species (ROS) into less harmful species. In this way, melatonin protects lipids, proteins and DNA from oxidative damage [6]. As a potent free radical scavenger, the antioxidant cascade of melatonin, i.e., melatonin and its secondary and tertiary metabolites, distinguishes it from other classic antioxidants. Given that the metabolites of melatonin, following its interaction with ROS, retain their ability to scavenge free radicals, one melatonin molecule has the capacity to scavenge up to ten ROS, this in contrast to the classic antioxidants that detoxify only one or less ROS [7]. In this way, melatonin can be hydroxylated by interaction with free radicals, something which causes an immediate intramolecular rearrangement when then leads to the formation of a third ring, thereby giving rise to a metabolite denominated as cyclic 3-hydroxymelatonin (c3OHM) (Figure 1). Likewise, c3OHM is also a potent free radical scavenger that can be converted by two ^•^OH to another key metabolite of melatonin, *N*^1^-acetyl-*N*^2^-formyl-5-methoxykynuramine (AFMK). At the same time, AFMK can be generated by direct conversion of melatonin, which comprises various enzymatic, pseudoenzymatic, free radical-mediated and photochemical mechanisms. Among these, the most relevant is the enzymatic conversion of melatonin to AFMK by indoleamine 2,3-dioxygenase (IDO) or myeloperoxidase (Figure 1). Finally, AFMK is easily deformylated to *N*^1^-acetyl-5-methoxykynuramine (AMK). To date, two enzymes capable of catalysing this reaction have been identified, arylamine formamidase and hemoperoxidase. Moreover, the formation of AMK from AFMK by ^•^OH has also been suggested. The deformylated product AMK appears to be a radical scavenger of considerably higher reactivity than AFMK because it easily undergoes single-electron transfer reactions, which can in turn generate further metabolites (Figure 1) (for an extensive review, see [7]).

On the other hand, melatonin is a pleiotropic molecule that is not only involved in the control of circadian rhythms but also participates in the regulation of the immune response, inhibition of carcinogenesis, proliferation of stem cells and modulation of aging, among other things [8]. Interestingly, this indolamine has been suggested as a promising agent for the management of reproductive disorders over the last two decades.

The interest in human reproduction has progressively grown over the years with the aim of generating new diagnostic and therapeutic tools to improve human fertility. However, it is a challenging subject of study due to population heterogeneity, ethical limits when experimenting with humans, high research costs and appropriate technologies [9]. The purpose of this article review is to summarize the current state of knowledge of the importance of melatonin in reproductive physiology and to provide a critical evaluation of the data available from existing studies on its potential uses in assisted reproductive techniques (ART) to improve fertility success rates.

## 2. Functions of Melatonin in Reproductive Physiology

### 2.1. Role of Melatonin in Sperm Physiology

Spermatogenesis is a testosterone-dependent event driving male gamete differentiation and maturation in the testis. However, mature spermatozoa are maintained in a quiescent state within the testis and must be therefore activated for a successful fertilization. The phenomenon of sperm activation is referred to as sperm capacitation, which is a multistep process involving changes in sperm form and function that are induced by extracellular structures of the oocyte. Such changes include initiation of motility, chemotaxis (i.e., swimming toward the oocyte in response to chemical concentration gradients), binding to the oocyte coat, the acrosome reaction (i.e., the release of hydrolytic enzymes from the acrosome), oocyte matrix penetration, and fusion of the two plasma membranes. Parallelly, reciprocal sperm-induced oocyte activation occurs with structural and metabolic changes in the oocyte that result in fertilization and which ultimately trigger embryo development (for a detailed review, see [10]. In this context, melatonin can pass through the blood–testis barrier and enter testicular cells. Moreover, the indoleamine acts via membrane melatonin receptors 1 (MT1) and 2 (MT2), which are G-protein-coupled receptors that control testosterone synthesis, and hence spermatogenesis, by regulating cyclic adenosine monophosphate (cAMP) transduction cascades [11].

Normal sperm function depends on low levels of ROS generation in order to promote the signal transduction pathways associated with capacitation, binding to the zona pellucida (i.e., the oocyte extracellular coat) and sperm chromatin condensation. When generated in excess, however, ROS can induce lipid peroxidation that, in turn, disrupts the membrane characteristics that are critical for the maintenance of sperm function, including the capacity to fertilize an egg [12]. Taking advantage of its outstanding antioxidant and free radical scavenger properties, melatonin reduces oxidative damage in mitochondria, DNA fragmentation, lipid peroxidation of plasma membrane and apoptotic markers in spermatozoa [13]. Nonetheless, this protection is not only due to its free radical scavenger properties but also to its action on the MT1 membrane receptor [14]. Furthermore, melatonin regulates sperm capacitation through the modulation of bicarbonate secretion and mobilization of intracellular calcium, which is dependent on the MT2 receptor [15].

### 2.2. Influence of Melatonin on Ovarian Follicle Development and Ovulation

Melatonin functions in female reproduction are based on its direct actions in the ovary [16]. The expression of MT1 and MT2 receptors in granulosa cells, luteal cells, antral follicles, and corpus luteum of rats indicates that it has essential roles in the regulation of mammalian reproductive processes. In fact, melatonin modulates granulosa cells steroidogenesis and follicular function in rodents and humans [17].

Folliculogenesis is the intricated process of ovarian follicle formation and is fundamentally dependent on circulating levels of FSH. The formation of ovarian follicles relies on low levels of ROS as well, as they act as second messengers modulating the expression of genes involved in oocyte maturation. Nevertheless, an excess of ROS may produce oxidative stress that can cause damage to granulosa and oocyte cells in the follicle [18]. For this reason, melatonin is essential for the maintenance of the pro-oxidant-antioxidant balance of the oocyte, thus protecting the female gamete from oxidative damage and regulating a healthy folliculogenesis [17,18]. Melatonin is found in follicular fluid at high concentrations (three-times higher than blood levels), where it stimulates granulosa cell proliferation through activation of mitogen-activated protein kinases (MAPK) [19]. Melatonin increases proportionally with the growth of the follicle [20] so that the larger the follicles the greater the concentration of melatonin. This high concentration of the indolamine during the pre-ovulatory phase is involved in the production of progesterone, which leads to luteinization and therefore to a successful ovulation [17].

### 2.3. Melatonin and Luteal Phase

Melatonin levels increase during the luteal phase as compared with the follicular phase, thus suggesting that the indolamine has a direct action in the modulation of this phase. Furthermore, melatonin binding sites as well as MT1 and MT2 receptors have been found to be expressed in human granulosa lutein cells, which is in line with the fact that melatonin stimulates progesterone release in human granulosa lutein cells [17,21]. On the other hand, melatonin also acts in the balance between luteotrophic and luteolytic regulators by inducing an increase of luteotrophic prostaglandin E_2_ (PgE2) and a reduction of the luteolytic modulator PgF2α [21].

In women suffering luteal phase defect, which is characterized by low blood flow and ROS-evoked oxidative stress, it has been observed that melatonin provides protection to granulosa lutein cells and increases progesterone production by corpus luteum via a reduction in oxidative stress [22].

### 2.4. Effects of Melatonin in the Placenta

The placenta synthesizes melatonin de novo and expresses melatonin MT1 and MT2 receptors, through which melatonin promotes placental cell survival [23]. Melatonin performs anti-apoptotic effects in the villous cytotrophoblasts, avoiding their excessive cell death, and acts as an antioxidant in the syncytiotrophoblasts. Furthermore, melatonin helps to maintain homeostatic processes in the placenta, which reduces the probability of pathologies such as preeclampsia [24]. Preeclampsia is a systemic maternal–foetal disorder characterized by hypertension after twenty weeks of gestation and is associated with maternal organ dysfunction and/or foetal growth restriction [25]. In preeclampsia, circulating levels of melatonin as well as its synthesis and receptor abundance are decreased [26]. For this reason, melatonin treatment could be potentially useful in this disorder, as suggested by Hannan et al. (2018), who have demonstrated that melatonin increased the expression of several antioxidant enzymes, including thioredoxin (TXN) in primary trophoblasts, placental explants and human umbilical vein endothelial cells (HUVEC), glutamate–cysteine ligase (GCLC) in placental explant tissue and HUVEC cells, and quinone acceptor oxidoreductase 1 (NQO1) in placental explant tissue [26].

### 2.5. Actions of Melatonin during Parturition

Melatonin levels increase with advancing gestation and reach their peak during labour, which suggests that the indolamine helps promote uterine contractions [27]. In fact, at the end of pregnancy, uterine contractions are stronger at night, when the concentration of circulating melatonin is the highest [28]. Interestingly, the myometrium expresses both oxytocin and melatonin MT2 receptors, whose expression are also increased during parturition. Melatonin and oxytocin trigger the same signalling pathway involving phospholipase C (PLC) and protein kinase C (PKC), which leads to the activation of myosin light chain kinase (MYLK) and ultimately promotes uterine muscle contractions. It has been demonstrated that melatonin acts synergistically with, and sensitizes uterine muscle to, oxytocin, thus producing its maximal contraction [29]. Moreover, melatonin increases the expression of protein connexin 43, which is necessary for myometrial cell communication and uterine contractions synchronization [27]. After childbirth, serum melatonin levels decrease rapidly [27].

### 2.6. Influence of Melatonin on Seasonal Reproduction

Melatonin is an important rhythmic hormone that regulates the circadian and seasonal rhythms of the body, thereby affecting the reproductive physiology of seasonally reproducing animals [30]. On one hand, seasonal reproduction assures that the offspring are delivered at the time of the year that maximizes their survival, which is mostly spring or early summer. Therefore, these species are reproductively mature when the day lengths are long with a brief nocturnal melatonin peak (e.g., hamster, horse). On the other hand, short day breeders (e.g., buffalo, sheep, deer) successfully breed during the winter when the duration of the nocturnal melatonin rise is prolonged. Importantly, in both long- and short-day breeding mammals, the annual cycle of reproductive competence and mating is regulated by the changing duration of the nocturnal melatonin peak [31]. Due to the important role of melatonin in the reproductive function of these animals, multiple studies have investigated the effect of exogenous melatonin out of the breeding season. Generally, findings of these studies have demonstrated that melatonin successfully improved sperm quality, as well as conception and fertilization rates in different animal species [32,33,34,35,36,37,38,39]. However, more investigations are necessary to give a definite recommendation on the use of melatonin as treatment to increase reproductive efficiency, thus allowing for increased productivity and flexibility of reproductive management. For an extensive review on this topic, the reader is encouraged to check the recent article [31] and the references therein.

## 3. Melatonin Application in Assisted Reproductive Techniques (ART)

In recent years, many studies have investigated the potential use of melatonin in ART to improve success rates. These techniques include oocyte manipulation, artificial insemination, in vitro fertilization (IVF), and embryo culture and transfer [40].

### 3.1. Sources of Reactive Oxygen Species (ROS) in ART

**Figure 2:**
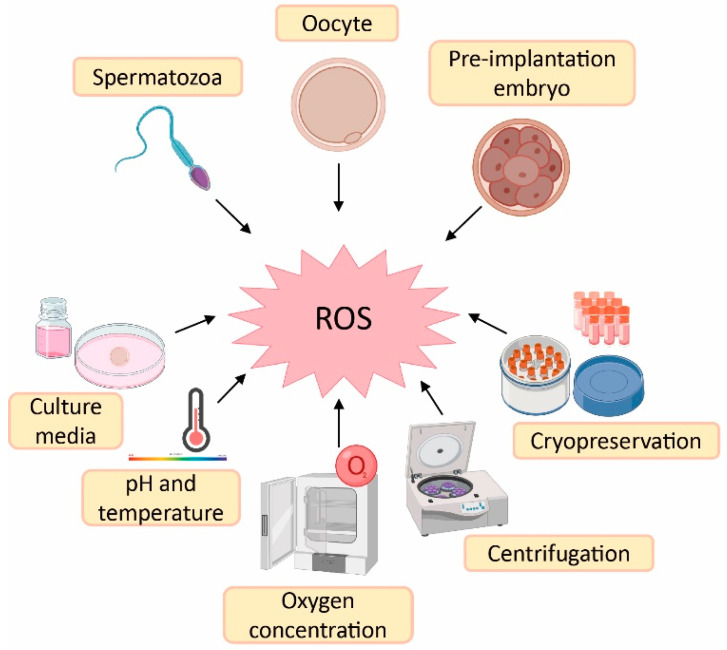
Sources of reactive oxygen species (ROS) during assisted reproductive techniques (ART). Created in BioRender.com.

Despite the physiological role of ROS on gamete structure and function, an exacerbated production of ROS could be detrimental for gamete physiology [12,18]. In this sense, there is a higher risk of oxidative stress during ART procedures compared with in vivo physiological conditions. The reason is the lack of physiological defence mechanisms and the presence of intrinsic sources of ROS such as oocytes, cumulus mass cells, spermatozoa and leukocytes [41]. Likewise, there are extrinsic factors responsible for ROS generation such as culture media, pH, temperature, oxygen concentration, centrifugation and cryopreservation [41,42] (Figure 2).

Culture media used during ART have an important impact on embryo quality and, consequently, on treatment success [41]. Some culture media contain metallic ions such as iron and copper. These ions lead to ROS generation, which implies that supplementation of the culture media with antioxidants could be beneficial to reduce ROS formation [43]. The maintenance of pH is also an important variable in culture media, as it influences sperm motility and its binding to oocyte, oocyte maturation and embryo development [44]. The maintenance of culture media pH is highly dependent on levels of CO_2_ and temperature, which should remain constant at 5% and 37 °C, respectively. Nevertheless, in the case of those procedures carried out outside an incubator, the pH is maintained by using culture media with reduced levels of bicarbonate or by including a pH buffer [43]. Furthermore, high atmospheric oxygen concentrations can influence embryo quality due to oxidative stress induction. This is the rationale behind the use of low atmospheric oxygen concentrations (5%) in some ART laboratories to mimic in vivo conditions—where others may use an oxygen concentration of 20% [45]. In fact, it has been demonstrated that an atmospheric oxygen concentration of 5% increases embryo quality, implantation and pregnancy rates, as well as live birth rates compared with an oxygen concentration of 20% [46].

Regarding spermatozoa preparation, centrifugation is a common step to separate spermatozoa from the seminal plasma and other components such as death cells, immature spermatozoa and leukocytes [43,47]. However, spinning sperm cells for more than 10 min leads to increased levels of ROS [42]. Furthermore, prolonged centrifugation times increase temperature of the sample, which also affects sperm motility [43]. Finally, cryopreservation is an ultra-low-temperature technique to maintain cells and tissues (from −80 °C to −196 °C) [48]. This method is the best option to preserve human gametes; however, freeze–thaw cycles dramatically increase ROS production and reduce antioxidant defences of spermatozoa, thus rendering them more sensitive to oxidative stress. At the same time, this oxidative stress leads to lipid peroxidation of the sperm membrane [49].

**Figure 3:**
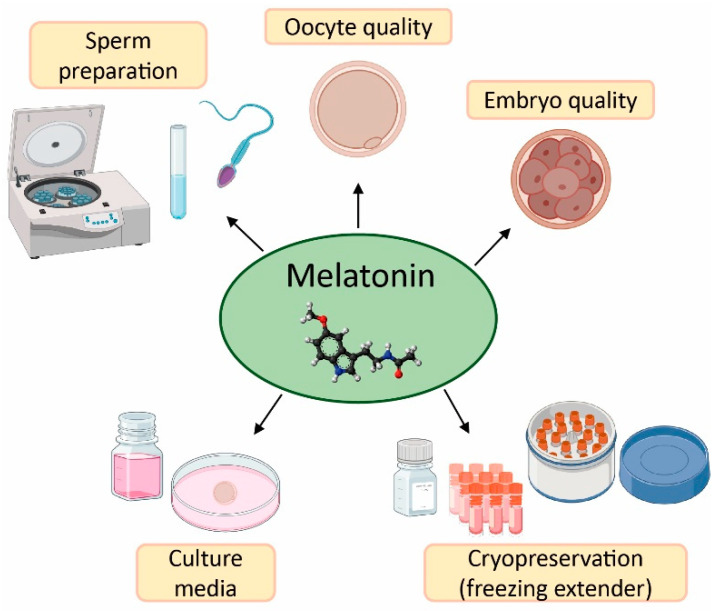
Applications and benefits of melatonin in assisted reproductive techniques (ART). Created in BioRender.com.

The field of reproductive medicine has achieved remarkable advances in the last few years. However, ongoing research is nowadays focused on enhancing the success rates of infertility treatments. For this purpose, in vitro and in vivo studies have concentrated their efforts on the application of antioxidants (e.g., vitamins C, D, E, resveratrol, and quercetin) [50,51,52,53,54,55], and particularly melatonin, in ART to counteract the negative effects of oxidative stress due to its free radical scavenging properties (Figure 3) [20,56].

### 3.2. Effect of Melatonin in Oocyte Quality and Embryo Quality

Numerous in vitro studies have supplemented culture media with melatonin so as to enhance oocyte maturation, oocyte fertilization and embryo development [20]. This approach assumes that oxidative stress accelerates apoptosis in oocytes and hence influences their capacity for fertilization. In fact, animal studies have shown that oxidative stress occurs after oocyte in vitro incubation for only 8 h; however, supplementation of oocyte culture media with 1 mM melatonin markedly relieved such a stress in a time-dependent manner in mouse oocytes, thereby delaying the onset of apoptosis. Moreover, melatonin supplementation also significant improved embryo quality [57]. The addition of 1 µM melatonin in oocyte culture media has also been proved with a prolonged in vitro incubation time of 52 h. The results demonstrate that the indoleamine was able to enhance the quality and development of porcine oocytes by decreasing ROS generation, apoptosis and DNA damage [58]. Similarly, low melatonin doses, i.e., 1 nM and 0.1 µM, improved the production and quality of bovine blastocysts, substantially increased the expression of important genes related to embryo development such as DNA methyltransferase 3a (DNMT3A), occludin (OCC) and cadherin (CDH1), and decreased the expression of aquaporin 3 (AQP3), which leads to an enhanced resistance to apoptosis [59,60].

Ovarian aging is characterized by a gradually depleted number of primordial follicles and a diminished quality of oocytes, thus causing a progressive reduction of fertility [61]. An investigation with 10-week-old female mice has demonstrated that the administration of water containing 100 µg/mL of melatonin delays ovarian aging. This supplementation, kept until mice were 43-weeks old, resulted in a higher number of primordial, primary and antral follicles, as well as better fertilization and blastocysts rates in treated mice in comparison with littermate control mice [62]. Additionally, melatonin significantly enhanced telomere length in old mice, and improved the expression of aging-related genes, such as sirtuins (SIRT1, SIRT3) and the autophagy-related gene microtubule-associated protein light chain 3 (LC3). Melatonin has also been shown to be able to upregulate 40 ribosome-related genes that are commonly downregulated during aging, these results demonstrating the capacity of melatonin to delay ovarian aging [62].

As for in vivo human studies, several trials have investigated the efficacy of melatonin administration to patients who underwent in vitro fertilization and embryo transfer (IVF-ET) procedure with the idea of rising follicular melatonin concentrations and hence improving oocyte quality [40]. In this sense, oral supplementation with 3 mg/day melatonin in women undergoing IVF-ET increased the percentage of mature oocytes and the number of top-quality embryos, although no significant differences were observed in fertilization rates and clinical pregnancy rates compared to a control group [63]. The same melatonin dosage was also tested in patients with poor oocyte and embryo quality and resulted in a better fertilization rate in the second cycle of IVF-ET in comparison with the first cycle without melatonin supplementation [64]. Other studies have been carried out in women with diminished ovarian reserve who received 3 mg/day melatonin commencing the fifth day of their menstrual cycle till the day of follicular puncture. The number of mature oocytes and top-quality embryos were higher in melatonin treated women than in the control group; however, no statistically significant differences were found in clinical pregnancy and spontaneous miscarriage rates between both groups [65]. In relation to unexplained infertility, oral supplementation with 3 mg/day or 6 mg/day of melatonin for 40 days rebalanced intrafollicular oxidative state, and enhanced oocyte quality and IVF success rates [66]. Furthermore, melatonin supplementation (5 mg/day) in IVF cycles was also effective in women aged over 40, and raised intrafollicular levels of indolamine, the number of mature oocytes and embryo quality [67]. Nevertheless, all these positive findings differ from other studies in which melatonin was unable to ameliorate oocyte quality. For example, in a clinical trial with oral administration of different antioxidants, including 0.975 mg of melatonin, there was an observed improvement in embryo quality upon melatonin treatment, but with no significant differences in terms of the number of follicles, mature oocytes and clinical pregnancy rates [68]. Similarly, doses of 2, 4 and 8 mg of melatonin administered twice a day enhanced neither the number of mature oocytes and embryos nor clinical pregnancy rates, even though the dose of 8 mg resulted in higher concentrations of intrafollicular melatonin compared with the placebo group [56].

As in female patients, melatonin administration was also studied in infertile men to investigate its effects on sperm quality and the quality of the embryos retrieved from their couples when undergoing an IVF cycle. The results demonstrate that supplementation for 45 days of 6 mg melatonin/day promoted a remarkable increase of seminal total antioxidant activity and a reduction in sperm DNA oxidative damage. Moreover, embryos obtained from women whose male couple was taking melatonin experienced significant increment in the percentage of grade A (top quality), B (good quality) and C (impaired quality) embryos, but a decrease in grade D (poor quality, not recommended for ET) embryos, according to the Spanish Association for the Study of Reproductive Biology (ASEBIR) criteria [69].

### 3.3. Application of Melatonin in Sperm Preparation for ART

Sperm preparation for ART aim at the selection and enrichment of motile and functionally competent spermatozoa from the ejaculate [70]. Starting from simple washing of spermatozoa, conventional techniques for the separation of spermatozoa from seminal plasma are based on different principles such as migration (which relies on the presence of motile spermatozoa within the semen sample, e.g., swim-up procedure), filtration (relies on sperm motility and the propensity of sperm to adhere to filtration matrices, e.g., glass wool filtration) and density gradient centrifugation (relies on sperm motility and the property of sperm to collect at the border between liquid phases, e.g., continuous density gradient with different media) [71]. Different studies carried out in diverse animal models have proved the effects of melatonin supplementation, at different doses, during sperm preparation [72,73,74,75,76,77,78,79]. In this regard, thawed bovine sperm samples were treated with 1 mM melatonin. The results demonstrate that the indolamine decreased the expression of pro-apoptotic genes such as caspase-3 and BAX and caused a dramatic rise in the expression of both the anti-apoptotic genes Bcl-2 and X-linked inhibitor of apoptosis protein (XIAP), and the antioxidant enzyme catalase (CAT). Likewise, in the same study, a concentration of 10 µM melatonin enhanced plasma membrane integrity and acrosome integrity, along with a reduction of the intracellular ROS levels [80]. The very same dose (10 µM) also proved to be efficient in sex-sorted bull semen as it protected semen samples against oxidative stress by increasing the activity of endogenous antioxidants such as Gpx, superoxide dismutase (SOD) and CAT, while inhibiting phosphatidylserine externalization and lipid peroxidation (measured as MDA levels), which are events related to apoptosis and acrosomal membrane integrity, respectively. Moreover, it was found that the dose of 10 µM melatonin led to an increment of the fertilization capacity and an enhancement in embryo development with respect to both the untreated control group and the different doses of the indolamine (1 nM, 0.1 µM and 1 mM) [81].

The role of melatonin in sperm capacitation has also been studied, this process being necessary for the sperm cells to acquire fertilizing capacity [82]. In this sense, low melatonin concentrations (100 pM) have been shown to modulate sperm capacitation by rising motile spermatozoa subpopulation in ram samples, thereby leading to better oocyte fertilization rates after IVF [83,84]. In fact, one of the first investigations reporting the involvement of melatonin in sperm motility modulation revealed that melatonin concentrations ranging from 1 pM to 1 µM, when added to supernatant after swim-up sperm selection, enhanced sperm hyperactivation, this action being dependent on MT1 receptor [85].

In relation to human studies, it has been observed that preincubation with 6 mM melatonin during sperm capacitation readily improved progressive motility and membrane integrity of asthenoteratozoospermic men samples [86]. Moreover, it has been reported that the use of lower doses, i.e., 1 mM melatonin, also produced good results since it provoked a significant increment of spermatozoa suitable for oocyte fertilization. Additionally, melatonin (1 mM) enhanced migration of spermatozoa with compacted DNA in oligozoospermic human samples and also avoided DNA fragmentation in normozoospermic human samples [87,88]. Likewise, a concentration of 2 mM melatonin also displayed beneficial effects in human sperm motility. Thus, the addition of melatonin after swim-up resulted in an increase in the number of fast and progressively motile spermatozoa, along with an improved sperm viability [89]. On the other hand, it has been demonstrated that a concentration of 1 mM melatonin exerted an anti-apoptotic effect in human spermatozoa treated with H_2_O_2_ or progesterone due to the inhibition of caspase-3 and the activation of caspase-9, and also prevented phosphatidylserine externalization, which is one of the main hallmarks of apoptosis [90]. Subsequent experiments have demonstrated that 1 mM melatonin managed to revert H_2_O_2_-induced DNA fragmentation and suggested that the protective effect of melatonin on sperm apoptosis is dependent on MT1 receptor and ERK signalling [14]. Interestingly, the fact that the indoleamine prevents DNA oxidative damage is not only dependent on its antioxidant effect but also relies on its ability to regulate diverse DNA repair pathways, including, but not limited to, base excision repair, homologous recombination and mismatch mediated repair, by different mechanisms (for a detailed review, see [91]).

### 3.4. Melatonin as Protective Agent in Gametes Cryopreservation

Sperm cryopreservation is the most commonly used method in cancer patients as they undergo aggressive treatments that can affect sperm quality and ultimately lead to azoospermia [92]. The main drawback of cryopreservation is that sperm quality can be negatively affected due to the freeze–thaw cycles, which may result in oxidative stress, lipid peroxidation increase and loss of plasma membrane integrity, hence disturbing the capacity of sperm–oocyte fertilization [93,94,95,96,97,98,99]. For this reason, the scientific community has focused its attention on the possible protective effects of melatonin on spermatozoa during cryopreservation given its powerful antioxidant action. This protection is associated with a reduction of lipid peroxidation events in sperm cells, which is related to the melatonin-evoked increase in both total antioxidant capacity and activity of antioxidant enzymes [100].

Several studies have proved the role of melatonin as an effective cryoprotectant in sperm cryopreservation. For instance, it has been reported that the supplementation with 2 mM or 3 mM melatonin in the semen extender counteracted the adverse effects of freeze–thaw cycles in bull sperm, as it lessened lipid peroxidation and boosted total antioxidant capacity and activity of antioxidant enzymes [101]. Similarly, the use of melatonin at a concentration of 100 μM or 1 mM improved both motility and viability parameters in cryopreserved buffalo semen samples, which was positively reflected in their in vitro fertilization capacity and the percentage of embryos obtained [102,103,104]. Moreover, it has also been documented that the addition of 1 mM melatonin in the cryoprotectant was efficient in various animal models such as rabbit, ram, horse, and dog. These studies proved that melatonin enhanced sperm DNA and acrosome integrity [105,106,107], which led to increased total cleavage rates and, hence, to higher fertilization and birth rates [105,108]. Furthermore, the indolamine managed to decrease oxidative stress by ameliorating antioxidant enzymes activation and, therefore, reducing ROS concentration during cryopreservation process [109]. Furthermore, the supplementation of the extender with 500 μM melatonin has been shown to improve the viability of post-thaw mouse sperm samples because of an increased expression of the anti-apoptotic gene B-cell lymphoma-extra-large Bcl-xL and a reduction in the percentage of viable spermatozoa with ROS overproduction [110].

On the other hand, studies carried out with human sperm samples have demonstrated that the use of 100 μM melatonin added as cryoprotectant was able to significantly raise sperm viability and membrane integrity, while diminishing intracellular ROS levels and lipid peroxidation. Of note, this supplementation did not have any detrimental effect on human sperm during cryopreservation [11,93]. Likewise, other authors have also reported that different melatonin concentrations (10 μM and 3 mM) resulted in higher viability and motility of cryopreserved spermatozoa, and lower intracellular ROS levels [111,112]. Finally, a recent study investigated the effect of 2 mM caffeine added before cryopreservation in normozoospermic semen samples previously treated with 2 mM melatonin. The findings showed that the combination of caffeine and melatonin ameliorated sperm motility and mitochondrial activity compared with samples treated only with melatonin [113].

Regarding mature oocyte cryopreservation, this technique can be used for women facing anticipated fertility decline for various reasons, including gonadotoxic cancer therapies, surgeries with risk of damage to ovary or oophorectomies, and women with increased risk of primary ovarian insufficiency [114,115,116]. With the idea of minimizing cellular osmotic and/or oxidative stresses during this procedure, the use of antioxidants such as melatonin has been implemented in the last few years. In this sense, it has been reported that loading porcine cumulus–oocyte complexes with melatonin plus glycine (1 μM and 6 mM, respectively) during vitrification (an ultra-rapid method of cryopreservation) enhanced the developmental competency of vitrified porcine oocytes, while lessening levels of ROS and apoptotic occurrence in mature oocytes [117]. Similarly, it has been demonstrated that the addition into vitrification media of melatonin and resveratrol (1 pM and 0.5 μM, respectively) co-encapsulated by solid lipid nanocarriers synergistically improved maturation, fertilization, and embryo development rates and decreased extra/intracellular ROS levels in mature oocytes [118]. Importantly, the indoleamine can also improve the effect of cryopreservation in human oocytes, as has been recently demonstrated [119]. Apart from protecting oocytes during cryopreservation, melatonin has also been proven to foster in vitro maturation of vitrified mouse [120,121,122,123,124,125,126,127,128,129] and equine [130] oocytes. Nevertheless, other studies found no effect of exogenous melatonin on development of cryopreserved oocytes in mouse [131].

Altogether, these studies indicate that melatonin can be used as an effective cryoprotectant, which would acquire special clinical relevance in cryopreserved samples from oncological patients that choose this method for preserving their fertility.

### 3.5. Impact of Melatonin on Reproductive Organs Pathophysiology

#### 3.5.1. Endometriosis

Endometriosis is associated with an exacerbated production of ROS due to an imbalance of oxidants and antioxidants and, therefore, the search for new treatments is focused on antioxidant therapy with the use of scavenging molecules such as melatonin [132].

In vitro studies with endometriotic epithelial cells derived from patients with endometriosis have shown that 1 mM of melatonin blocked 17β-estradiol-induced migration, invasion and epithelial–mesenchymal transition (EMT) through the upregulation of the Numb endocytic adaptor protein (Numb) and the low activity of the neurogenic locus notch homolog protein 1 (Notch1) [133].

On the other hand, a large number of animal studies have investigated the potential use of melatonin in endometriosis. Thus, an intraperitoneal injection of 10 mg melatonin/kg/day for 28 days in a rat model of surgically induced endometriosis caused volume and weight reduction of the implants via modulation of the expression of vascular endothelial growth factor (VEGF), which is involved in angiogenesis, and tissue inhibitor of metalloproteinase-2 (TIMP-2), which is significantly decreased in women with endometriosis [134,135]. Similar results have been observed with different doses of melatonin. For instance, the administration of 20 mg melatonin/kg/day for two weeks in surgically induced endometriotic rats produced a greater regression of endometriotic foci than that observed with letrozole, which is used to treat endometriosis, and the recurrence rate was also lower after the cessation of treatment compared with letrozole [136]. Other studies have shown that the dose of 20 mg melatonin/kg/day was more efficient than 10 mg/kg/day, causing a higher regression of endometriotic lesions and a significant decrease of MDA in an oophorectomized rat endometriosis model and in a severe combined immunodeficient (SCID) mice endometriosis model [137,138,139]. Additionally, a higher dose of melatonin (48 mg/kg/day) administered for 10–20 days in ovariectomized mice promoted apoptosis in the endometriotic tissue mediated by a reduction of the anti-apoptotic B-cell lymphoma 2 gene (Bcl-2) and an increase of the proapoptotic Bcl-2-associated X protein (BAX) and caspase-9. Moreover, melatonin suppressed metalloproteinase-3 (MMP-3) expression, whose activity is associated with the formation of endometriotic lesions at an early stage [140].

The potential use of melatonin for endometriosis has also been investigated in a phase II, randomized, double-blind, placebo-controlled trial. Interestingly, daily oral supplementation with 10 mg of melatonin for eight weeks reduced daily chronic pelvic pain scores (39.80%) and dysmenorrhea (38.01%) in patients with endometriosis. Melatonin also improved sleep quality and diminished the levels of brain-derived neurotrophic factor (BDNF), which is related to the pathogenesis of chronic pain, independently of pain levels (Table 1) [141]. Nevertheless, oral administration of 10 mg/day of melatonin was ineffective as an analgesic in women with dysmenorrhea but without signs of endometriosis (Table 1) [142], contrary to the results obtained after administering 3 mg melatonin /day, which reduced pain and enhanced subjective sleep in women with primary dysmenorrhea (Table 1) [143]. While some previous findings lend support to the use of indoleamine [134,135,136,137,138,139,140,141], new clinical trials are warranted to convincingly demonstrate the effectiveness of melatonin as a possible pharmacological and/or adjuvant treatment in the management of endometriosis.

#### 3.5.2. Polycystic Ovary Syndrome (PCOS)

Polycystic ovary syndrome (PCOS) is a common endocrine disorder that causes hyperandrogenism and infertility due to a dysfunctional follicular maturation and anovulation. Numerous animal studies have focused on the use of melatonin to investigate its putative effects on oocyte quality in PCOS. In this sense, the addition of 10 µM melatonin in the culture media of oocyte cumulus obtained from a PCOS female mouse model enhanced oocyte quality due to the lowered presence of free radicals, which resulted in increased fertilization rates [144]. A recent study using the same animal model and melatonin concentration showed that the indoleamine promoted an increment in both oocyte maturation-related genes, such as growth differentiation factor-9 (Gdf9) and bone morphogenetic protein 5 (Bmp5), and antioxidant enzymes, such as glutathione peroxidase 1 (Gpx1) and superoxide dismutase 1 (Sod1), compared to untreated group [145]. Furthermore, there was an observed anti-apoptotic effect of melatonin mediated by a decline of the expression of BAX and a rise of anti-apoptotic Bcl-2. Finally, this study demonstrated an inverse correlation between levels of ROS and concentration of the indolamine in the culture media in the melatonin-treated groups [145].

As for human studies, melatonin supplementation of in vitro culture medium has been evaluated in PCOS patients undergoing IVF-ET. The results demonstrate that the addition of 10 µM melatonin to in vitro maturation media enhanced embryo implantation and pregnancy rates with respect to non-supplemented control group [146]. In relation with the utilization of the indoleamine as oral pharmacological treatment in PCOS patients, the findings of some trials are encouraging. First, the administration of 2 mg melatonin/day for six months to PCOS patients enhanced menstrual irregularities in 95% of patients and waned levels of androgens and anti-Mullerian hormone, whose basal levels are above the normal range in PCOS patients. Concurrently, FSH levels, which are shrunk in this syndrome, were significantly enlarged compared with a control group (Table 1) [147]. The effects of melatonin oral supplementation were also investigated in PCOS patients undergoing intrauterine insemination [148] and in women with PCOS undergoing an IVF-ET cycle [149]. In these trials, the synergistic effect of 3 mg melatonin/day and 4000 mg myoinositol/day remarkably elevated the number of mature oocytes and grade I embryos with respect to an untreated control group and myoinositol-treated group, although no significant differences were found in pregnancy rates (Table 1).

On the other hand, the combination of 3 mg of melatonin with 250 mg of magnesium also proved to be effective in PCOS patients since it enhanced sleep quality and decreased serum testosterone and insulin levels, which are usually high in women with PCOS (Table 1) [150].

Given the safety profile of the indoleamine, and despite the necessity of larger clinical studies to confirm melatonin effectiveness, some fertility clinics have already included melatonin as a supplement in IVF protocols, especially, in the case of PCOS patients.

#### 3.5.3. Varicocele

Varicocele is a common clinical disease in andrology that has detrimental effects on semen quality, sperm function, and pregnancy outcomes in some men [151]. Most importantly, oxidative stress seems to have a central role in the pathogenesis of varicocele-induced infertility [151]. In this context, the role of melatonin in the prevention of testicular damage was first investigated in animal model of experimentally induced varicocele [152,153]. Such reports found that melatonin administration at a dose of 10 mg/kg/day antagonized the activation of germ cell apoptosis evoked by varicocele, which was attributed to the prevention of oxidative lipid damage. In the same line, more recent investigations have revealed that the antioxidant/anti-apoptotic efficacy of melatonin treatment of varicocele rats is mediated by microRNA-34a/SIRT1/forkhead transcription factors-class O (type1) (FOXO1) signal transduction pathway [154].

Though there are no studies on the therapeutical potential of melatonin on varicocele patients, low levels of melatonin in semen have been observed in infertile men with varicocele [155]. Likewise, one study has recently investigated the impact of melatonin supplementation on semen quality and total antioxidant capacity after varicocelectomy of infertile male patients [156], thus concluding that melatonin therapy adds an extra benefit to varicocelectomy by improving sperm concentration, motility, and morphology as well as total antioxidant capacity.

#### 3.5.4. Ovarian Cancer

A plethora of new drugs has been proposed as adjuvant therapeutic strategies for ovarian cancer management, including melatonin. This is due to its antiproliferative, anti-angiogenic, pro-apoptotic and immunomodulatory properties [157]. In fact, in vitro studies have demonstrated the anti-tumour effect of 800 µM melatonin for 72 h, as it is able to inhibit tumour growth through the downregulation of cyclin-dependent kinases 2 and 4 (CDK2 and CDK4) in ovarian cancer cell lines PA-1 and OVCAR-429 [158]. Similarly, a concentration of 3.4 mM melatonin has been shown to hinder proliferation and migration by 23% in cancer stem cells derived from ovarian cancer cell line SK-OV-3. Moreover, melatonin (3.4 mM) also decreased the expression of EMT-related proteins such as zinc finger E-box-binding homeobox 1 and 2 (ZEB1 and ZEB2), snail, and vimentin, and increased the levels of E-cadherin, a negative regulator of EMT [159]. On the other hand, melatonin may enhance the therapeutic effect of cisplatin in ovarian cancer as it has been proven that, in cisplatin-treated SK-OV-3 cells, the indolamine further promoted cytotoxicity and apoptosis through caspase-3 activation and through suppression of the extracellular signal-regulated kinase (ERK)/90-kDa ribosomal S6 kinase (p90RSK)/heat shock protein 27 (HSP27) cascade, thereby improving cisplatin-induced apoptosis [160].

Regarding animal studies, the anti-tumour effect of melatonin in an ovarian cancer-induced rat model has been investigated. Melatonin, injected at a dose of 200 µg/100 g body weight/day for 60 days, reduced ovarian tumour mass by 20%, synchronized the oestrous cycle and forestalled the incidence of sarcomas, endometrioid carcinomas and cystic teratomas [161]. Furthermore, the same dose of melatonin may modulate different molecular events associated with ovarian cancer in rat models. Thus, melatonin has been shown to be involved in apoptosis induction as it upregulates of pro-apoptotic proteins (i.e., p53, BAX and caspase-3), downregulates the anti-apoptotic protein Bcl-2 and strengthens DNA fragmentation in an in vivo model of ovarian cancer [162]. Likewise, melatonin treatment has also been effective in modulating the angiogenic signalling pathway by lowering the expression of several angiogenic factors such as transforming growth factor β-1 (TGFβ1), VGEF and vascular endothelial growth factor receptor 2 (VEGFR2) [163]. Additionally, melatonin has been shown to be able to suppress the increased expression levels of proteins involved in ovarian cancer signalling, including epidermal growth factor receptor 2 (Her-2), p38 mitogen-activated protein kinase (p38 MAPK), protein kinase B (AKT), mammalian target of rapamycin (mTOR) [164], and toll-like receptor 4 (TLR4) [165].

As for human studies, a phase II study has investigated the effectiveness of a combination of melatonin with tamoxifen as therapy in patients with untreatable metastatic solid tumours, wherein two ovarian cancer patients were enrolled. Drugs were given orally at a daily dose of 20 mg of tamoxifen in the midday and 20 mg of melatonin at bedtime. The first ovarian cancer patient, who had lung metastasis, did not respond to the treatment, and survived for only four months. However, in the second ovarian cancer patient, who had lung and lymph nodes metastasis, the disease was stabilized, and a 10-month survival was achieved (Table 1) [166]. This trial demonstrates the potential of melatonin as a therapeutic agent in ovarian cancer, although further clinical assays are necessary to draw definitive, unequivocal conclusions.

**Table 1:** Melatonin clinical trials on ovarian pathologies.

Pathology	Patient Population	Dosage of Melatonin	Main Outcomes	Reference
Endometriosis	Patients with endometriosis	10 mg/day × 8 weeks	Reduced daily chronic pelvic pain scores and dysmenorrhea Improved sleep quality Decreased levels of BDNF	Schwertner et al. [141]
	Women with dysmenorrhea but without endometriosis	10 mg/day during menstrual week	Ineffective analgesic effect	Söderman et al. [142]
		3 mg/day during menstrual period	Reduced pain Enhanced subjective sleep	Keshavarzi et al. [143]
PCOS	PCOS patients	2 mg/day for 6 months	Enhanced menstrual irregularities in 95% patients Decreased androgens and anti-Mullerian levels Enlarged FSH levels	Tagliaferri et al. [147]
	PCOS patients undergoing intrauterine insemination	3 mg/day from day 3 of menstruation until day of hCG administration	Augmented follicles size and endometrial thickness Improved clinical pregnancy rates	Mokhtari et al. [148]
	PCOS patients undergoing IVF-ET	3 mg MEL/day + 4000 mg myo-inositol/day	Elevated the number of mature oocytes and grade I embryos	Pacchiarotti et al. [149]
	PCOS patients	3 mg MEL + 250 mg magnesium	Enhanced sleep quality Decreased serum testosterone and insulin levels	Alizadeh et al. [150]
Ovarian cancer	Ovarian cancer patients	20 mg MEL + 20 mg tamoxifen	Patient survival partially improved	Lissoni et al. [166]

BDNF: brain-derived neurotrophic factor; FSH: follicle stimulating hormone; hCG: human chorionic gonadotropin; IVF-ET: in vitro fertilization-embryo transfer; MEL: melatonin; PCOS: polycystic ovary syndrome.

## 4. Conclusions

As impaired oxidative balance appears to explain fertility treatment failure, research on reproductive medicine has focused on developing efficient antioxidant therapies to bypass such an issue. In this scenario, melatonin has emerged in the last few years as a valuable tool. Thus, the addition of melatonin to culture media helps gametes to efficiently fight against oxidative stress in so far as the indoleamine reportedly improves oocyte and sperm quality during gametes preparation and/or in vitro culture. Furthermore, melatonin also acts as an effective cryoprotectant by counteracting cellular osmotic and/or oxidative stresses that occur during cryopreservation of spermatozoa and oocytes. More importantly, both animal research and clinical trials have provided evidence that oral supplementation with melatonin prevents oxidative stress-induced damage in vivo, which enhances gametes quality and may eventually lead to higher fertility success rates. This intervention is particularly relevant in the management of diverse ovarian pathologies since melatonin supplementation has been informed to be beneficial in animal models of, and patients with endometriosis, PCOS and/or ovarian cancer.

From a medical perspective, melatonin has increasingly gained attention in recent years due to scientific evidence supporting its potential therapeutical use in reproductive medicine and its safety profile. On one hand, most fertility clinics recommend the use of dietary supplements to aid fertility as they may exert positive effects on different fertility targets, such as hormonal balance, ovulation, gametes quality or embryo quality, and hopefully on the likelihood of achieving pregnancy. In this sense, several dietary supplements for male and female fertility that contain melatonin are currently being marketed in Europe and the USA (e.g., Gestagyn men, Seidivid, FertyBiotic, Ovosicare Fertility, or Theratonin). Additionally, some fertility clinics have already included melatonin as antioxidant therapy in IVF protocols, especially, in the case of PCOS patients. Nonetheless, further clinical studies are warranted to better understand the proper dose, timing, and application of melatonin to enhance fertility.

## Data Availability

Not applicable.
